# A TRANSITIONS OF CARE INTERVENTION (FOR OLDER ADULTS) TO REDUCE 30-DAY READMISSIONS FROM SUBACUTE REHABILITATION

**DOI:** 10.1093/geroni/igae098.1813

**Published:** 2024-12-31

**Authors:** Rebecca Slossberg, Lucrezia Renzetti, Edith Burns, Raj Patel, Nazish Ilyas, Alexander Trost, Alexander Rimar

**Affiliations:** Northwell Health, New York, New York, United States; Northwell Health, New York, New York, United States; Northwell Health, Manhasset, New York, United States; Northwell Health, New York, New York, United States; NYU Langone Health, New York, New York, United States; Northwell Health, New York, New York, United States; Northwell Health, New York, New York, United States

## Abstract

Hospital readmissions for older adults from subacute rehabilitation facilities (SAR) are a common challenge for healthcare systems. Patient medical complexity, lack of communication during transitions of care, and other factors contribute to otherwise avoidable readmissions. We describe a pilot intervention of Geriatrics and Palliative (GAP) consultation after hospital discharge with the goal of reducing avoidable readmissions from SAR. 136 patients seen by the GAP team in 2023 during a first hospitalization were included. The GAP Nurse Practitioner conducted SAR consultations on a convenience sample of 85 patients (Intervention group, IG), with the remaining 51 acting as non-intervention control (CG). Overall patients in the IG group were not significantly different from the CG based on age, sex, race, LOS, or LACE score. Mean age was 86.8 (SD=7.44 years), majority female (62.5%) and white (67.2%). Readmission rate was 18.8% for IG vs. 27.4% for CG, a 38.7% lower odds of readmission for IG. Readmitted patients from IG were similar to CG readmits (age, sex, LACE) but had longer index LOS (8.76 + 10.2 vs. 6.16 + 4.2 days, t=2.06, p=0.041). Cost analysis showed a 25% cost savings for Star IG vs CG. Mean total cost was highest for CG Star and lowest for CG non-star. Results suggest that following patients in transition to SAR post hospital discharge may help reduce avoidable readmissions in older adults by ensuring continuity of communication. Further research to understand what elements of follow-up care impacted readmission (e.g., hospice eligibility, pain levels, code status, etc.,) is needed.

